# Do Perioperative Antibiotics Improve Outcomes After Hypospadias Repair? A Systematic Review and Meta-Analysis of Pediatric Literature

**DOI:** 10.3390/children13020194

**Published:** 2026-01-30

**Authors:** Maria Escolino, Maria Sofia Caracò, Valerio Mazzone, Mustafa Azizoglu, Giovanni Esposito, Mauro Porcaro, Marco Castagnetti, Ciro Esposito

**Affiliations:** 1 Division of Pediatric Surgery, Federico II University Hospital, 80131 Naples, Italy; mariasofia.caraco@unina.it (M.S.C.); valerio.mazzone@unina.it (V.M.); ciroespo@unina.it (C.E.); 2 Department of Pediatric Surgery, Basaksehir Cam and Sakura City Hospital, Istanbul 34480, Turkey; mustafa.azizoglu@saglik.gov.tr; 3 Department of Stem Cell and Tissue Engineering & 3D Bioprinting, Istinye University, Istanbul 34010, Turkey; 4 CEINGE Advanced Biotechnologies, 80131 Naples, Italy; 5 Division of Pediatric Radiology, Federico II University Hospital, 80131 Naples, Italy; mauro.porcaro@unina.it; 6 Department of Pediatric Urology, Bambino Gesù Children Hospital, 00165 Rome, Italy

**Keywords:** antibiotics, hypospadias, hypospadias repair, antibiotic prophylaxis, postoperative complications

## Abstract

**Highlights:**

**What are the main findings?**

- Perioperative antibiotics do not significantly reduce postoperative complications after hypospadias repair.
- No clear benefit was observed for preoperative, postoperative, or combined antibiotic regimens.

**What are the implications of the main findings?**

- Routine antibiotic use in hypospadias surgery may be unnecessary.
- A selective, risk-based antibiotic strategy supports antimicrobial stewardship without compromising outcomes.

**Abstract:**

**Background/Objectives**: The role, timing, and duration of antibiotic therapy in hypospadias repair remain controversial, with substantial variability in clinical practice and a lack of evidence-based guidelines. This systematic review and meta-analysis aimed to evaluate whether preoperative, postoperative, or combined perioperative antibiotic regimens influence postoperative outcomes after hypospadias repair. **Methods**: A systematic literature search of PubMed, MEDLINE, Scopus, Embase, and Web of Science was conducted in accordance with PRISMA guidelines to identify studies published between 2000 and 2025 that reported on perioperative antibiotic administration in pediatric patients undergoing hypospadias surgery. Three comparisons were assessed: (i) postoperative antibiotics versus no antibiotics, (ii) preoperative antibiotics versus no antibiotics, and (iii) combined pre- and postoperative antibiotics versus no antibiotics. Outcomes included infectious complications, wound dehiscence, urethrocutaneous fistula, meatal or urethral stenosis, and other postoperative complications. Random-effects meta-analyses were performed, with pooled odds ratios reported together with 95% confidence intervals. **Results**: Ten studies comprising a total of 9493 patients were included. Perioperative antibiotic use was not associated with a significant reduction in infectious complications (OR 0.95, 95% CI 0.63–1.44; *p* = 0.81), urethrocutaneous fistula (OR 1.89, 95% CI 0.87–4.12; *p* = 0.10), or wound dehiscence (OR 1.52, 95% CI 0.98–2.35; *p* = 0.06) compared with no antibiotic use. Preoperative antibiotic prophylaxis alone did not result in a reduction in infectious complications or wound dehiscence. Combined pre- and postoperative antibiotic therapy did not demonstrate a clear benefit over no antibiotics in terms of infectious complications, although available data were very limited. **Conclusions**: Routine perioperative antibiotic therapy does not significantly reduce postoperative complications after hypospadias repair. These findings support a selective, risk-based approach to antibiotic use rather than routine administration in hypospadias surgery. Further well-designed prospective studies are needed to establish evidence-based perioperative antibiotic protocols in pediatric hypospadias surgery.

## 1. Introduction

Hypospadias is one of the most common congenital anomalies of the male genitalia, and surgical repair represents the definitive treatment to restore urethral function and achieve satisfactory cosmetic outcomes [1,2,3]. Although advances in surgical techniques and perioperative care have improved outcomes over time, postoperative complications—including infectious complications, wound dehiscence, urethrocutaneous fistula (UCF), and meatal or urethral stenosis—remain a significant clinical concern [4,5]. Their incidence varies widely across published series and is influenced by multiple factors, including hypospadias severity, surgical technique, tissue handling, urinary diversion, and perioperative management [6,7,8,9,10]. Perioperative antibiotic therapy is commonly employed in hypospadias surgery with the aim of reducing postoperative infections and improving surgical outcomes. However, despite its widespread use, substantial variability exists in clinical practice regarding the indication, timing, and duration of antibiotic administration [11,12]. Strategies range from single-dose preoperative prophylaxis to prolonged postoperative antibiotic therapy, or even to the complete omission of antibiotics in selected cases. This heterogeneity reflects the lack of clear, procedure-specific, evidence-based recommendations [13,14]. Given the widespread but variable use of perioperative antibiotics in hypospadias repair, the absence of clear evidence-based guidelines, and the heterogeneous and sometimes conflicting results reported in the literature, a systematic evaluation of the available evidence is warranted. In this context, a comprehensive synthesis of the existing evidence is needed to clarify whether routine perioperative antibiotic therapy is justified in hypospadias surgery or whether a more selective and individualized approach should be preferred.

This systematic review and meta-analysis aimed to evaluate whether antibiotic administration—preoperative, postoperative, or combined pre- and postoperative—influences postoperative outcomes after hypospadias surgery, compared with alternative or no antibiotic regimens.

## 2. Materials and Methods

This systematic review was registered on PROSPERO (CRD420251266161) and conducted in accordance with the Preferred Reporting Items for Systematic Reviews and Meta-analyses (PRISMA) guidelines. A full study protocol was written and uploaded to PROSPERO (https://www.crd.york.ac.uk/PROSPERO/view/CRD420251266161 and accessed on 15 December 2025).

### 2.1. Search Strategy

A comprehensive electronic literature search was conducted across the PubMed, Scopus, MEDLINE, Embase and Web of Science databases to identify studies reporting on perioperative antibiotic administration in pediatric patients undergoing hypospadias surgery. The final database search was conducted on 30 September 2025. The search strategy adopted the following keywords consistent with MeSH terms: (antibiotic prophylaxis OR anti-bacterial agents OR antibiotics) AND (hypospadias OR urethral repair OR urethroplasty OR postoperative complications OR urethrocutaneous fistula OR wound infection OR wound dehiscence) AND (humans OR children OR pediatrics). The search covered a 25-year period (from 1 January 2000 to 31 March 2025) to ensure a comprehensive identification of all relevant studies on perioperative antibiotic use in hypospadias repair. The reference lists of relevant studies were manually reviewed to identify additional eligible studies, and duplicate records were excluded. The full electronic search strategies for all databases are provided in the Supplementary Table S1.

### 2.2. Study Selection

Study selection was conducted independently by three reviewers (MSC, VM, and ME). After removal of duplicate records, titles and abstracts were independently screened by two reviewers (MSC and VM), followed by full-text assessment. A third reviewer (ME) verified the consistency of study selection. Disagreements were resolved through discussion or, when necessary, by consultation with a senior author (CE). Inter-rater reliability during the abstract screening phase was evaluated using Cohen’s kappa coefficient, which demonstrated substantial agreement (κ = 0.79). The full texts of potentially eligible studies were subsequently assessed in detail according to the predefined inclusion and exclusion criteria. Study eligibility was defined following the PICOS framework:

- Population: Pediatric patients (age ≤ 18 years) diagnosed with hypospadias undergoing surgical repair.
- Intervention: Preoperative antibiotic therapy, postoperative antibiotic therapy, or combined pre- and postoperative antibiotic therapy.
- Comparison/Control: No antibiotic therapy or alternative perioperative antibiotic regimens.
- Outcome(s): Infectious complications [bacteriuria, urinary tract infection (UTI), wound infection (WI), surgical site infection (SSI)], wound dehiscence, UCF, meatal/urethral stenosis, and other postoperative complications.
- Study design: Comparative clinical studies including randomized controlled trials (RCTs), prospective cohort studies, and retrospective studies. Only English-language publications were considered eligible for inclusion.

Exclusion criteria were studies with unavailable full texts, studies focused on adult patients (>18 years) undergoing hypospadias repair or patients undergoing urethral reconstruction for conditions other than hypospadias, studies exclusively including patients with complex disorders of sex development or associated major urogenital anomalies requiring staged or non-standard reconstruction and studies with incomplete outcome data or not available in English. We also excluded case reports, small case series, reviews, editorials, conference abstracts, and non–peer-reviewed articles.

### 2.3. Data Extraction

Data were extracted using a predefined standardized spreadsheet (Microsoft Excel, version 15.4). Extracted variables included author and publication details, study design, and patient characteristics (sample size, age, hypospadias severity, surgical technique, and type and duration of urinary diversion), as well as antibiotic regimens (no antibiotics, preoperative antibiotics alone, postoperative antibiotics alone, or combined pre- and postoperative antibiotics), and reported postoperative outcomes. For continuous variables, median values with interquartile ranges were extracted when available. Data extraction was independently performed by three reviewers (MSC, VM, and ME) using the standardized form to ensure consistency. Any discrepancies were resolved through discussion and consensus, with arbitration by a senior author (CE) when required. Inter-rater reliability for the data extraction process was assessed and demonstrated a high level of agreement (κ = 0.81).

### 2.4. Study Endpoints

The primary endpoints comprised variables related to antibiotic regimens, including the type, modality and duration of administration. Secondary endpoints included postoperative complications following hypospadias repair, namely infectious complications, UCF, wound dehiscence, meatal or urethral stenosis, and other postoperative complications as defined by the individual studies. Infectious complications were defined as a composite outcome including bacteriuria, UTI, WI, and SSI, according to the study-specific definitions.

The present systematic review was designed to address the following key research questions: (1) Does perioperative antibiotic therapy reduce postoperative complications compared with no antibiotic use? (2) Is there a difference in outcomes between preoperative antibiotics alone and combined pre- and postoperative antibiotics? (3) Does combined pre and postoperative antibiotic therapy provide additional benefit compared with postoperative antibiotics alone?

### 2.5. Risk of Bias Assessment

The methodological quality of the included studies was evaluated using the Newcastle-Ottawa Scale (NOS). Quality assessment was performed independently by three reviewers (MSC, VM and ME), with any discrepancies resolved through discussion. Interobserver agreement was high across all assessed domains.

### 2.6. Statistical Analysis

Statistical analysis was performed using Review Manager (RevMan) software, version 5.4. For dichotomous outcomes, effect estimates were expressed as odds ratios (ORs) with corresponding 95% confidence intervals (CIs), while mean differences were reported for continuous variables. Fisher’s exact test was used for comparisons of categorical variables, and a *p* value ≤ 0.05 was considered statistically significant. Reporting bias was evaluated through visual inspection of funnel plots and, when at least three comparative studies were available, formally assessed using Egger’s regression asymmetry test. A *p*-value < 0.05 was considered indicative of small-study effects. Ethical approval was not required, as this study was a systematic review based exclusively on previously published data.

### 2.7. Management of Heterogeneity

Given the expected clinical and methodological heterogeneity among the included studies—related to differences in study design, hypospadias severity, surgical techniques, antibiotic regimens, and outcome definitions—specific strategies were adopted to manage heterogeneity in the quantitative synthesis. A random-effects model was applied for all meta-analyses to account for between-study variability. The degree of statistical heterogeneity across the included studies was formally assessed using the χ^2^ test and quantified with the I^2^ statistic, with values > 50% considered indicative of substantial heterogeneity. To further explore potential sources of heterogeneity, predefined subgroup analyses were conducted according to the timing of antibiotic administration, including: (1) postoperative antibiotics alone versus no antibiotics, (2) preoperative antibiotics alone versus no antibiotics, and (3) combined pre- and postoperative antibiotics versus no antibiotics. These subgroup analyses were selected a priori based on clinically relevant differences in antibiotic strategies and were performed when at least two comparative studies were available for pooling. In addition, outcomes were analyzed separately to avoid inappropriate pooling of clinically heterogeneous endpoints. When the number of studies was insufficient to support formal subgroup or sensitivity analyses, results were interpreted descriptively and with appropriate caution.

### 2.8. Certainty of Evidence Assessment

A formal certainty-of-evidence assessment (e.g., GRADE approach) was not performed. Given the substantial clinical and methodological heterogeneity across the included studies, the predominance of observational study designs, and the limited number of studies available for several comparisons, a formal grading of evidence certainty was considered unlikely to provide meaningful or reliable estimates. Results were interpreted by integrating effect sizes, CIs, consistency across analyses, and the risk of bias of the included studies.

## 3. Results

### 3.1. Study and Patient Characteristics

In total, 94 records were identified through the initial database search. After removal of duplicate records (*n* = 4) and clearly irrelevant studies (*n* = 20), 70 abstracts were screened. Of these, 47 records were excluded because they consisted of systematic reviews, meta-analyses, narrative reviews, books, or conference abstracts. The remaining 23 full-text articles were assessed for eligibility, and an additional 13 were excluded for not meeting the predefined inclusion criteria. Ultimately, 10 studies were deemed eligible and included in the systematic review [15,16,17,18,19,20,21,22,23,24].

**Figure 1:**
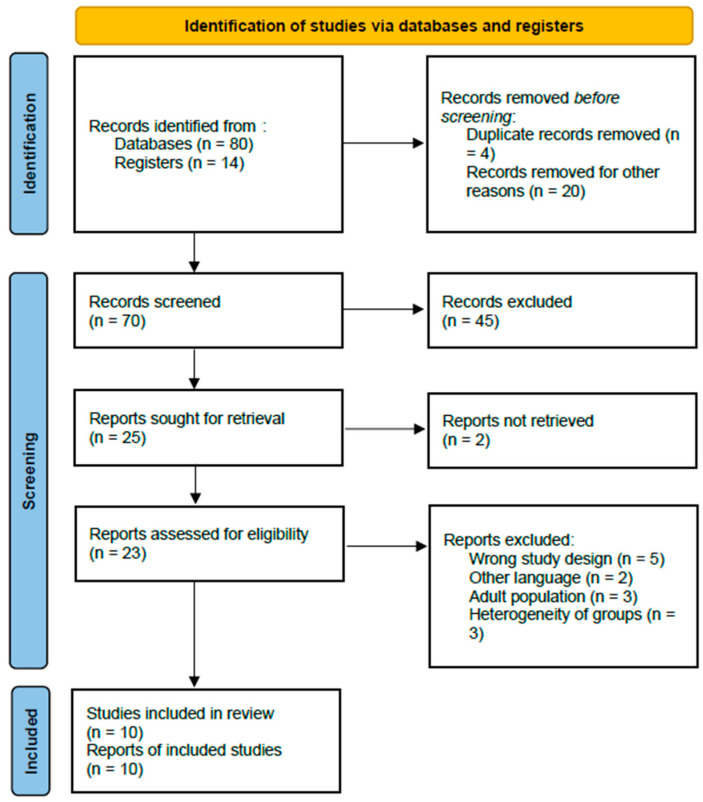
PRISMA flow chart of selection of publications for inclusion in the systematic review.

A detailed PRISMA flow chart illustrating the study selection process is presented in Figure 1.

The review included two prospective cohort studies, three retrospective comparative studies, one prospective randomized clinical trial (RCT), one prospective randomized controlled study, one randomized double-blinded placebo-controlled study, one retrospective cross-sectional study, and one retrospective cohort study.

Overall, 9493 male patients undergoing hypospadias surgery were included in the selected studies. The median age at the time of surgery was 12.57 months (range 6–168). The distribution of patients according to hypospadias severity, surgical technique, type and duration of postoperative urinary diversion, and antibiotic regimen is summarized in Table 1.

### 3.2. Methods Quality Assessment

Overall, the methodological quality of the included studies ranged from moderate to good. Most studies demonstrated adequate selection of study groups, clearly defined cohorts of pediatric patients undergoing hypospadias repair, and well-described antibiotic strategies. Comparability between groups was acceptable in most studies; however, adjustment for potential confounders—such as hypospadias severity, operative technique, and type or duration of urinary diversion—was inconsistently reported, particularly in retrospective studies. Outcome assessment was generally well defined, with most studies reporting clinically relevant postoperative endpoints, including infectious complications, UCF, wound dehiscence, and meatal or urethral stenosis. Follow-up duration was sufficient to capture early and intermediate postoperative complications in most series, although long-term outcomes were not uniformly addressed. Randomized and prospective studies achieved higher NOS scores than retrospective studies, primarily due to better comparability and more standardized outcome assessment. A detailed overview of the NOS scoring for each included study is provided in Table 2.

### 3.3. Antibiotic Strategies

Antibiotic strategies varied widely across the included studies with respect to timing, type, and duration of administration (Table 1). When used, preoperative antibiotic prophylaxis mostly consisted of a single intravenous dose administered at anesthesia induction, typically first- or second-generation cephalosporins (e.g., cefazolin or cefonicid), or clindamycin in patients with penicillin allergy. In several studies, preoperative prophylaxis was uniformly administered to all patients undergoing hypospadias repair, regardless of hypospadias severity or surgical technique.

Postoperative antibiotic therapy was equally heterogeneous. The most frequently prescribed agents included trimethoprim–sulfamethoxazole (TMP-SMX), trimethoprim (TMP) alone, cephalexin, amoxicillin or amoxicillin–clavulanate, nitrofurantoin, and, less commonly, fluoroquinolones or clindamycin. Postoperative antibiotics were generally administered orally and were often continued for the entire duration of urethral stenting or catheterization, with reported durations ranging from 1 postoperative day to 10 days or until stent removal.

Several studies [15,16,17,20,21,22] compared combined pre- and postoperative antibiotic regimens with either preoperative prophylaxis alone or postoperative therapy alone. In contrast, other studies [18,19,20,22,23,24] specifically evaluated postoperative antibiotic therapy versus no antibiotic use, particularly in distal or midshaft hypospadias repairs.

### 3.4. Postoperative Outcomes

Infectious complications were reported in 162/9493 (1.7%) patients and included bacteriuria (60/9493, 0.6%), complicated UTI (86/9493, 0.9%), WI and/or SSI (12/9493, 0.1%), cellulitis or infected inclusion cyst (4/9493, 0.04%). Urethroplasty specific complications were reported in 316/9493 (3.3%) patients, including UCF (78/9493, 0.8%), wound dehiscence (209/9493, 2.2%) and meatal or urethral stenosis (29/9493, 0.3%). Other postoperative complications occurred in 14/9493 (0.1%) subjects. The category of “other postoperative complications” was heterogeneous and inconsistently defined across studies. Reported events included urethral diverticulum, meatal regression, and adverse drug reaction (ADR), each occurring at very low frequencies. These outcomes were not uniformly reported and were often grouped differently across studies, limiting direct comparability.

When perioperative antibiotic use was compared with no antibiotic use, no significant differences were observed for infectious complications (OR 0.95, 95% CI 0.63–1.44; *p* = 0.81), UCF (OR 1.89, 95% CI 0.87–4.12; *p* = 0.10), wound dehiscence (OR 1.52, 95% CI 0.98–2.35; *p* = 0.06), or meatal/urethral stenosis (OR 0.89, 95% CI 0.34–2.34; *p* = 0.82). A statistically significant reduction was observed only for other postoperative complications (OR 0.33, 95% CI 0.11–1.00; *p* = 0.04).

Compared with preoperative antibiotics alone, combined pre- and postoperative antibiotic therapy did not reduce infectious complications (OR 1.15, 95% CI 0.71–1.89; *p* = 0.57) and was associated with higher risks of UCF (OR 7.08, 95% CI 4.16–12.07; *p* < 0.05), meatal/urethral stenosis (OR 6.05, 95% CI 2.45–14.92; *p* < 0.05), and other complications (OR 26.78, 95% CI 2.99–239.81; *p* < 0.05), while wound dehiscence was significantly reduced (OR 0.34, 95% CI 0.17–0.66; *p* = 0.001).

When combined pre- and postoperative antibiotics were compared with postoperative antibiotics alone, a significant reduction in UCF (OR 0.43, 95% CI 0.23–0.82; *p* = 0.008) and other complications (OR 0.25, 95% CI 0.06–1.02; *p* = 0.04) was observed, whereas no significant differences were found for infectious complications, wound dehiscence, or meatal/urethral stenosis. Although statistical significance was observed for “other postoperative complications” in selected analyses, these findings should be interpreted with caution. The very low number of events, heterogeneity in outcome definitions, and marginal CIs substantially limit the robustness and clinical relevance of this result. Accordingly, this outcome should be considered exploratory rather than indicative of a meaningful protective effect of perioperative antibiotic therapy.

All postoperative outcomes reported in each study are detailed in Table 3.

Across the included studies, substantial clinical heterogeneity was observed with respect to hypospadias severity (distal, midshaft, or proximal), surgical techniques, duration and type of urinary diversion, and antibiotic regimens in terms of agent, timing, and duration.

This non-random allocation of antibiotic strategies introduces a significant risk of confounding by indication, which likely influenced some of the observed associations. In particular, the paradoxical findings showing higher rates of UCF and meatal/urethral stenosis in patients receiving combined antibiotic therapy should be interpreted considering the clinical context and do not imply a causal adverse effect of antibiotics. Rather, combined pre- and postoperative antibiotic regimens were more frequently administered to patients undergoing more complex hypospadias repairs or considered at higher baseline risk of complications, including proximal hypospadias, redo surgery, longer urethral reconstruction, or prolonged urinary diversion.

Although some subgroup comparisons showed statistically significant differences, these findings should be interpreted with caution. Several of these analyses were based on a limited number of studies, low event rates, and wide CIs, which may reduce their clinical reliability. Comparisons involving combined pre- and postoperative antibiotic therapy versus other regimens are susceptible to selection bias and confounding by indication, as more intensive antibiotic strategies were often preferentially adopted in patients perceived to be at higher surgical risk. Therefore, these subgroup results should be considered exploratory and hypothesis-generating rather than definitive evidence of a causal effect.

### 3.5. Meta-Analyses

#### 3.5.1. Postoperative Antibiotics Alone vs. No Antibiotics

**Figure 2:**
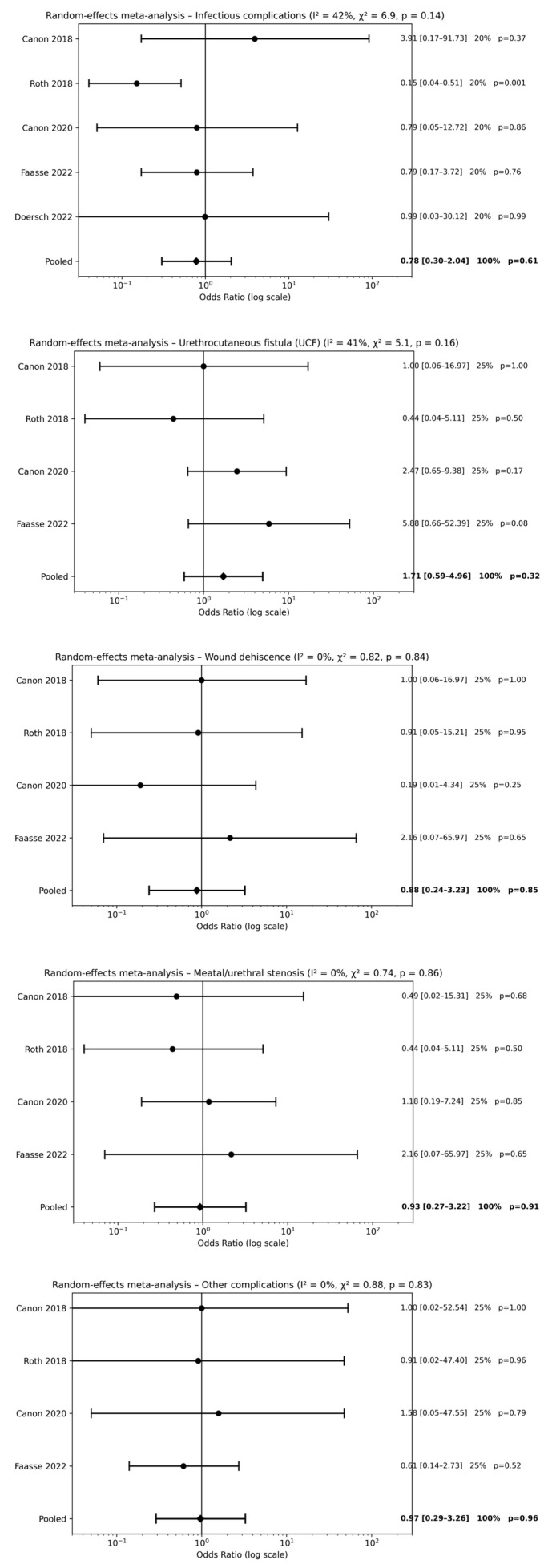
Forest plots comparison between postoperative antibiotics alone vs. no antibiotics on surgical outcomes [18,19,20,22,23].

Five studies [18,19,20,22,23] evaluated postoperative antibiotic therapy alone compared with no antibiotic use following hypospadias repair. Meta-analysis did not demonstrate a significant reduction in postoperative complications associated with postoperative antibiotic use. No statistically significant differences were observed for infectious complications [pooled OR 0.78, 95% CI 0.30–2.04; *p* = 0.61], UCF [pooled OR 1.71, 95% CI 0.59–4.96; *p* = 0.32], wound dehiscence [pooled OR 0.88, 95% CI 0.24–3.23; *p* = 0.85], meatal/urethral stenosis [pooled OR 0.93, 95% CI 0.27–3.22; *p* = 0.91], or other postoperative complications [pooled OR 0.97, 95% CI 0.29–3.26; *p* = 0.96] (Figure 2). Overall, effect estimates were consistently close to unity across all outcomes, indicating that postoperative antibiotic therapy alone does not provide a clinically meaningful benefit.

#### 3.5.2. Preoperative Antibiotics Alone vs. No Antibiotics

**Figure 3:**
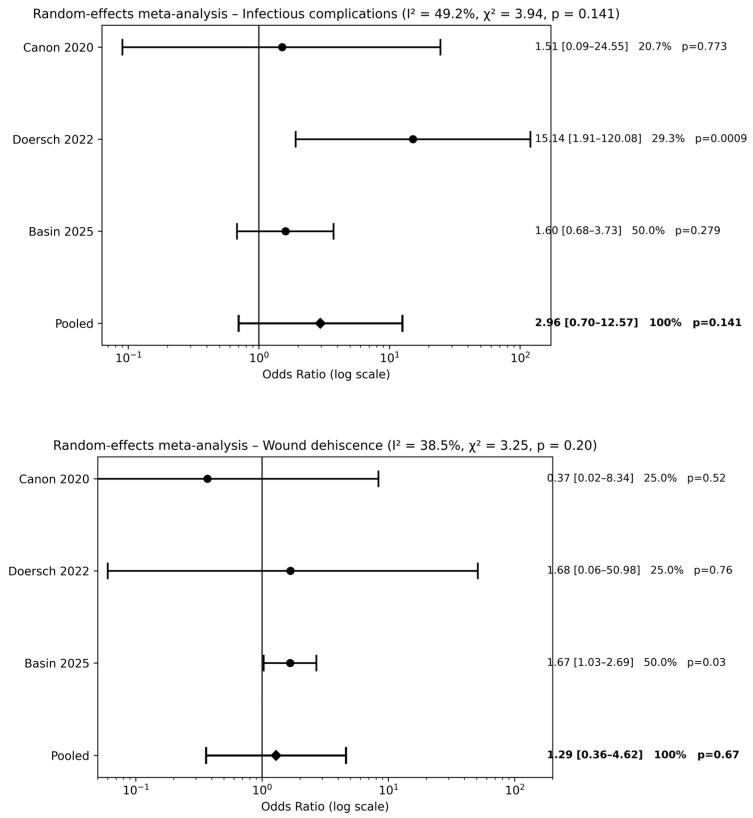
Forest plots comparison between preoperative antibiotics alone vs. no antibiotics on surgical outcomes (references [20,22,24]).

Three studies [20,22,24] evaluated preoperative antibiotic prophylaxis alone compared with no antibiotic use. Meta-analysis did not demonstrate a significant reduction in postoperative complications for infectious complications [pooled OR 2.96, 95% CI 0.70–12.57; *p* = 0.14] or wound dehiscence [pooled OR 1.29, 95% CI 0.36–4.62; *p* = 0.67] (Figure 3). Across outcomes, pooled effect estimates were close to unity, and CIs consistently crossed the line of no effect, indicating the absence of a measurable protective effect of preoperative antibiotic prophylaxis alone compared with no antibiotics.

#### 3.5.3. Combined Pre- and Postoperative Antibiotics vs. No Antibiotics

**Figure 4:**
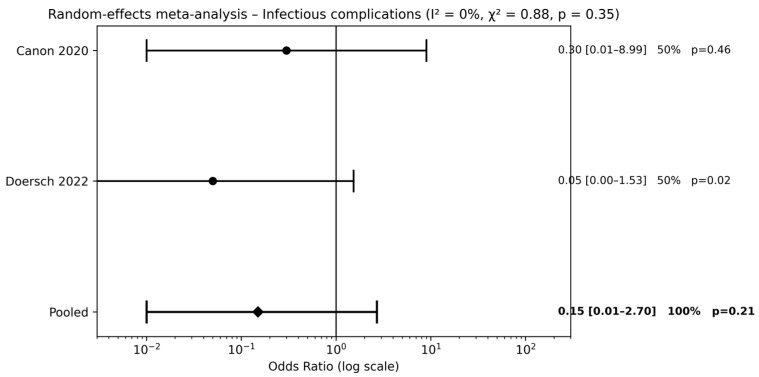
Forest plots comparison between combined pre- and postoperative antibiotics vs. no antibiotics on surgical outcomes (references [20,22]).

Two studies [20,22] compared combined pre- and postoperative antibiotic therapy with no antibiotic use. Meta-analysis did not demonstrate a significant reduction in infectious complications (pooled OR 0.15, 95% CI 0.01–2.70; *p* = 0.21) (Figure 4). The wide CIs indicate substantial uncertainty around the estimated effect size.

### 3.6. Publication Bias

Publication bias was assessed for outcomes with at least three comparative studies available. Visual inspection of funnel plots did not reveal significant asymmetry for any postoperative outcome when comparing postoperative antibiotics alone with no antibiotics (Supplementary Figure S1). Egger’s regression test did not demonstrate evidence of small-study effects for infectious complications (*p* = 0.12), UCF (*p* = 0.60), wound dehiscence (*p* = 0.67), meatal/urethral stenosis (*p* = 0.98), or other postoperative complications (*p* = 0.27).

For the comparison between preoperative antibiotics alone and no antibiotics, funnel plot inspection did not suggest relevant asymmetry for infectious complications or wound dehiscence (Supplementary Figure S2). Egger’s regression test was not significant for infectious complications (*p* = 0.98) or wound dehiscence (*p* = 0.23).

In the comparison between combined pre- and postoperative antibiotics and no antibiotics, formal assessment of publication bias was limited by the small number of studies included. Funnel plot inspection for infectious complications did not show obvious asymmetry (Supplementary Figure S3); however, Egger’s regression test was not applicable due to the inclusion of fewer than three studies. Overall, although no clear evidence of publication bias was identified, the limited number of studies for some outcomes warrants cautious interpretation of these findings.

No formal assessment of certainty of evidence was conducted for individual outcomes. The overall confidence in the findings was therefore based on the consistency of pooled estimates, width of CIs, risk of bias, and the degree of clinical and methodological heterogeneity across studies.

## 4. Discussion

The rationale for antibiotic use in hypospadias surgery has traditionally been extrapolated from general principles of SSI prevention and from adult urology practice [25,26,27,28]. However, hypospadias surgery presents several unique characteristics, including a pediatric patient population, delicate tissue planes, frequent use of urethral stents or catheters, and potential wound contamination with urine or stool in non-continent patients. Importantly, many of the most relevant postoperative complications following hypospadias repair—such as UCF and meatal stenosis—are multifactorial in origin and may be more closely related to surgical technique, tissue ischemia, and wound healing than to infection alone [5,6,7,29,30,31,32,33]. Consequently, the true clinical benefit of perioperative antibiotic therapy in this setting remains uncertain.

Over the past two decades, several studies have explored the relationship between perioperative antibiotic use and postoperative outcomes after hypospadias repair. Nevertheless, the available literature is characterized by marked heterogeneity in study design, patient populations, hypospadias severity, surgical techniques, antibiotic regimens, and outcome definitions [12,13,14]. As a result, published findings are inconsistent and sometimes conflicting, with some studies suggesting a reduction in selected complications, while others report no significant benefit or even potential adverse associations [34,35,36]. This substantial variability in antibiotic protocols underscores the lack of standardized perioperative antibiotic guidelines in hypospadias surgery and highlights the need for evidence-based recommendations. At the same time, increasing concerns regarding antimicrobial resistance and antibiotic-related adverse effects emphasize the importance of avoiding unnecessary antibiotic exposure, particularly in pediatric patients [36,37,38,39,40,41].

In this context, the present systematic review and meta-analysis aimed to clarify the role of perioperative antibiotic therapy in hypospadias repair by addressing three clinically relevant questions: whether perioperative antibiotics reduce postoperative complications compared with no antibiotics; whether combined pre- and postoperative antibiotic therapy provides additional benefit over preoperative antibiotics alone; and whether combined therapy offers advantages over postoperative antibiotics alone.

The most clinically relevant finding of this review is that perioperative antibiotic use does not result in a significant reduction in the most common postoperative complications following hypospadias repair. Across pooled analyses, no significant differences were observed for infectious complications, UCF, wound dehiscence, or meatal/urethral stenosis when perioperative antibiotics were compared with no antibiotic use. The only statistically significant finding was a reduction in other postoperative complications, an infrequently reported and heterogeneous outcome, which substantially limits its clinical interpretability.

These findings challenge the widespread assumption that routine antibiotic administration is necessary to prevent complications after hypospadias repair. Importantly, the overall incidence of infectious complications across the included studies was low (1.7%), suggesting that hypospadias surgery—particularly distal and midshaft repairs—may not carry a sufficiently high infection risk to justify routine antibiotic use. From a pathophysiological perspective, many key complications following hypospadias repair are not primarily driven by infection. UCF, meatal stenosis, and wound dehiscence are influenced by tissue quality, surgical technique, local ischemia, suture tension, and urinary diversion, rather than bacterial contamination alone [42,43,44,45]. Therefore, the lack of a protective effect of antibiotics observed in this meta-analysis appears biologically plausible. When focusing specifically on preoperative antibiotic prophylaxis, the meta-analysis demonstrated no significant benefit over no antibiotic use for infectious complications or wound dehiscence. Although preoperative antibiotics are often extrapolated from general surgical prophylaxis principles, their utility in hypospadias surgery remains questionable. Moreover, pooled estimates for preoperative prophylaxis were characterized by wide CIs, reflecting limited sample sizes and heterogeneity among studies. This uncertainty further supports the notion that routine preoperative prophylaxis may not be universally required, particularly in uncomplicated distal repairs performed under standardized sterile conditions.

Similarly, the comparison between combined pre- and postoperative antibiotic therapy and no antibiotics failed to demonstrate a significant reduction in infectious complications. The limited number of available studies and wide CIs precluded definitive conclusions. Interestingly, when combined therapy was compared with preoperative or postoperative antibiotics alone, conflicting results emerged. Combined therapy was associated with a reduction in wound dehiscence in one comparison and with lower UCF rates in another, but also with higher risks of selected adverse outcomes, including fistula and stenosis, when compared with preoperative antibiotics alone.

The findings of this meta-analysis are consistent with previous systematic reviews focusing on postoperative antibiotics in distal hypospadias repair, which similarly reported no clear benefit of prolonged antibiotic prophylaxis [12,13,14]. However, the present study expands upon prior work by including a broader range of antibiotic strategies, surgical techniques, and hypospadias degrees, as well as by evaluating preoperative and combined regimens. Taken together, the available evidence suggests that routine perioperative antibiotic therapy should not be considered mandatory for all patients undergoing hypospadias repair. Instead, a selective and individualized approach appears justified, reserving antibiotic use for patients with specific risk factors such as proximal hypospadias, redo surgery, prolonged urinary diversion, or documented preoperative infection. This strategy may reduce unnecessary antibiotic exposure, minimize ADR, and contribute to antimicrobial stewardship efforts.

Interpretation of these findings must account for the substantial clinical heterogeneity among the included studies. Hypospadias severity, surgical technique, use and duration of urinary diversion, and antibiotic protocols varied widely, reflecting real-world practice but also introducing important sources of bias. Although a random-effects model was appropriately applied, residual confounding cannot be fully excluded, particularly given the predominance of retrospective study designs. Statistically significant findings emerging from selected subgroup analyses should therefore not be overinterpreted. In the setting of limited sample sizes, low event rates, and wide CIs, such findings may be clinically misleading if considered in isolation. The observed associations, particularly those involving combined antibiotic regimens, are strongly influenced by confounding by indication and underlying case complexity. Confounding by indication is particularly relevant in this context. In several studies, combined pre- and postoperative antibiotic regimens were preferentially administered to patients with more complex clinical profiles, including proximal hypospadias, longer urethral reconstructions, redo surgery, or prolonged catheterization. These patients inherently carry a higher baseline risk of UCF, stenosis, and other complications. Consequently, the observed higher rates of UCF and meatal/urethral stenosis in patients receiving combined antibiotic therapy should not be interpreted as evidence of a causal adverse effect of antibiotics. Rather, these associations reflect underlying case complexity and surgical risk.

Caution is also warranted when interpreting the findings related to “other postoperative complications,” which represented a heterogeneous composite outcome with inconsistent definitions across studies. Although statistical significance was observed in some analyses, this result was driven by very low event rates, wide and marginal CIs, and outcomes of limited clinical relevance. These factors substantially weaken the reliability of this finding and preclude any meaningful inference regarding the effectiveness of perioperative antibiotic therapy based on this outcome alone.

The predominance of distal and midshaft hypospadias repairs in the included studies likely contributed to the very low overall incidence of infectious complications observed in this meta-analysis. This low baseline risk reduces the likelihood of detecting a measurable protective effect of antibiotics and limits the generalizability of the findings to proximal or redo hypospadias repairs, where operative complexity and baseline complication risk are substantially higher. In these higher-risk subgroups, a selective antibiotic strategy may be appropriate and warrants further investigation in future prospective studies.

Systemic perioperative antibiotic prophylaxis represents only one component of a broader infection prevention strategy in hypospadias surgery. Emerging evidence suggests that additional antimicrobial measures may also contribute to reducing infectious complications after hypospadias repair. A recent large 10-year single-center cohort study including 550 pediatric patients reported a significantly lower incidence of SSIs and postoperative UCF when triclosan-coated polydioxanone sutures were used compared with uncoated sutures [46]. Triclosan-coated materials have been shown to inhibit bacterial colonization and biofilm formation at the surgical site, providing a localized antimicrobial effect that may complement systemic antibiotic prophylaxis [47,48,49,50,51]. Although these findings are encouraging, the available evidence is largely observational and derives from single-center experiences. Well-designed prospective multicenter studies are therefore needed to clarify the role of such adjunctive strategies and to determine whether their routine use should be recommended in hypospadias surgery.

Several limitations of this study should be acknowledged. First, the included studies were heterogeneous in terms of design, patient populations, hypospadias severity, surgical techniques, antibiotic regimens, and outcome definitions, which may have influenced pooled estimates. Second, many studies were retrospective and lacked adequate adjustment for confounding variables, increasing the risk of selection bias and confounding by indication. Third, the number of studies available for certain comparisons—particularly combined pre- and postoperative antibiotics versus no antibiotics—was small, limiting statistical power and precluding robust assessment of publication bias. Fourth, most studies focused on short- to intermediate-term outcomes, and long-term complications were not consistently reported. Finally, antibiotic-related adverse effects and microbiological outcomes were inconsistently documented, preventing a comprehensive assessment of the risk–benefit balance of antibiotic use.

## 5. Conclusions

This systematic review and meta-analysis demonstrate that routine perioperative antibiotic therapy does not significantly reduce postoperative complications following hypospadias repair. No clear benefit was observed for preoperative antibiotics, postoperative antibiotics, or combined regimens when compared with no antibiotic use across the most clinically relevant outcomes. These findings support a more selective and individualized approach to antibiotic administration in hypospadias surgery rather than routine use. Antibiotic therapy should be considered on a case-by-case basis, considering patient- and procedure-specific risk factors such as hypospadias severity, redo surgery, prolonged urinary diversion, or the presence of preoperative infection. Well-designed prospective randomized studies with standardized surgical and antibiotic protocols are needed to establish robust evidence-based guidelines for perioperative antibiotic management in pediatric hypospadias repair and to identify patient subgroups that may benefit from targeted antibiotic strategies.

## Figures and Tables

**Table 1:** Summary of study features and patient demographics.

Author/Year	Study Design	Series, *n*=	Patient Age, (Months) Median (Range)	Hypospadias Degree (*n*=)	Operative Technique (*n*=)	Urinary Diversion Type (*n*=)	Urinary Diversion Duration, Median Days (Range)	No ATB (*n*=)	Pre-op ATB (*n*= and Type)	Postop ATB (*n*= and Type)	Postop ATB Duration, Days (Range)
Meir 2004 [15]	Prospective cohort study	52	n/a	Glanular (6) Coronal (27) Penile (16) Penoscrotal (3)	TIPU (52)	6–8 Fr silicon catheter (52)	8.6	n/a	Cefonicid (52)	Cephalexin (52)	1 POD up to 2 days after stent removal
		49	n/a	Glanular (3) Coronal (27) Penile (17) Penoscrotal (2)	TIPU (49)	6–8 Fr silicon catheter (52)	8.3	n/a	Cefonicid single dose (49)	n/a	n/a
Kanaroglu 2013 [16]	Prospective cohort study	78	12.72 (7.2–168)	Glanular (10) Coronal (47) Midshaft (12) Proximal (9)	TIPU + redo TIPU (67) Glanular approx. (6) Staged preputial flap/graft (5)	8 Fr silicon catheter (78)	7 (1–12)	n/a	Cefazolin single dose (78)	TMP (78)	1 POD for stenting duration
		71	17.28 (6–144)	Glanular (16) Coronal (39) Midshaft (8) Proximal (8)	TIPU + redo TIPU (60) Glanular approx (4) Staged preputial flap/graft (7)	8 Fr silicon catheter (71)	7 (2–14)	n/a	Cefazolin single dose (71)	n/a	n/a
Zeiai 2016 [17]	Retrospective cohort study	58	23.8	Penile (45) Penoscrotal (13)	TIPU (58)	Bladder catheter (58)	7	n/a	TMP-SMX single-dose (58)	TMP/SMX (58)	1 POD up to 3–7 days after stent removal
		55	17.7	Penile (45) Penoscrotal (10)	TIPU (55)	Bladder catheter (55)	7	n/a	TMP-SMX single-dose (55)	n/a	n/a
Roth 2018 [18]	Prospective Randomized clinical trial	35	9.8 (±3.1)	Midshaft (2) Megameatus (6) Distal shaft (27)	TIPU (17) Mathieu (4) MAGPI (7) Pyramid repair (6) Urethral advancement (1)	6Fr kendall-type urethral stent (35)	8 (6–10)	n/a	n/a	TMP-SMX (35)	6–10
		32	10.5 (±4.7)	Midshaft (2) Megameatus (4) Distal shaft (26)	TIPU (18) Mathieu (4) MAGPI (4) Pyramid repair (4) Urethral advancement (2)	6Fr kendall-type urethral stent (32)	8 (6–10)	32	n/a	n/a	n/a
Canon 2018 [19]	Retrospective randomized comparative study	24	8.4	Glanular (4) Coronal (7) Distal (13)	n/a	6–8 Fr urethral catheter (24)	7.1	n/a	n/a	TMP-SMX (22) Nitrofurantoin (1) Cephalexin (1)	7.1
		24	10.8	Glanular (3) Coronal (7) Distal (14)	n/a	6–8 Fr urethral catheter (24)	7.9	24	n/a	n/a	n/a
Canon 2021 [20]	Retrospective comparative study	96	10.5	n/a	TIPU (82) Duplay (9) MAGPI (3)	Urethral catheter (96)	6.5 (5–8)	96	n/a	n/a	n/a
		159	14.6	n/a	TIPU (116) Duplay (29) MAGPI (9) Barcatt (2) GAP (1)	Urethral catheter (159)	6.5 (5–8)	n/a	Cefazolin or Clindamycin if penicillin allergy (159)	TMP-SMX or Nitrofurantoin/Cefalexin (159)	6.5 (5–8)
		64	12	n/a	TIPU (47) Duplay (3) MAGPI (2) GAP (2)	Urethral catheter (64)	6.5 (5–8)	n/a	Cefazolin or Clindamycin if penicillin allergy (64)	n/a	n/a
		122	14.5	n/a	TIPU (96) Duplay (21) MAGPI (4)	Urethral catheter (64)	6.5 (5–8)	n/a	n/a	TMP-SMX or Nitrofurantoin/Cefalexin (122)	6.5 (5–8)
Manchanda 2023 [21]	Prospective randomized controlled study	16	n/a	n/a	n/a	Urethral catheter	7.5 (5–10)	n/a	Ceftriaxone (16)	Ceftriaxone iv for 48 h, followed by oral Amoxiclav (16)	Stenting duration 7.5 (5–10)
		24	n/a	n/a	n/a	Urethral catheter	7.5 (5–10)	n/a	Ceftriaxone (24)	n/a	n/a
Doersch 2022 [22]	Retrospective comparative study	67	11.4	n/a	Duplay (10) MAGPI (8) TIPU (49) Redo (2)	Urethral stent	5 (3–7)	67	n/a	n/a	n/a
		647	9.1	n/a	Duplay (75) MAGPI (17) Mathieu (5) Other (6) TIPU (544) Redo (33)	Urethral stent	5 (3–7)	n/a	Cefazolin (634) Ampicillin (3) Cefoxitin (1) Ceftriaxone (1) Clindamycin (11) Gentamycin (3) Vancomycin (1)	Amoxicillin (12) Amoxi-clav (2) Cephalexin (276) Ciprofloxacin (1) Clindamycin (1) Nitrofurantoin (2) TMP (3) TMP-SMX (350)	Stenting duration 5 (3–7)
		80	11.8	n/a	Duplay (64) MAGPI (9) Mathieu (5) Other (2) Redo (2)	Urethral stent	5 (3–7)	n/a	Cefazolin (76) Clindamycin (4)	n/a	n/a
		34	10.2	n/a	Duplay (20) MAGPI (13) Mathieu (1) Redo (3)	Urethral stent	5 (3–7)	n/a	n/a	Amoxicillin (3) Amoxi-clav (1) Clindamycin (4) TMP-SMX (25)	Stenting duration 5 (3–7)
Faasse 2022 [23]	Randomized double-blinded placebo-controlled study	48	10 (8–12)	Glanular (2) Coronal (19) Distal shaft (22) Mid-shaft (5)	TIPU (41) Duplay (4) MAGPI (1) Other (2)	Urethral stent	8 (6–9)	48	n/a	n/a	n/a
		45	10 (8–11)	Glanular (2) Coronal (19) Distal shaft (19) Mid-shaft (5)	TIPU (41) Duplay (2) MAGPI (1)	Urethral stent	8 (6–9)	n/a	n/a	TMP-SMX (45)	10
Basin 2025 [24]	Retrospective cross-sectional study	1221	12 (7.2–24)	n/a	1-stage distal hypo repairs (956) 1-stage proximal hypo repairs and second-third stage urethroplasty (147) Redo (118)	n/a	n/a	1221	n/a	n/a	n/a
		6392	14.4 (8.4–30)	n/a	1-stage distal hypo repairs (4070) 1-stage proximal hypo repairs and second-third stage urethroplasties (1281) Redo (1041)	n/a	n/a	n/a	Single dose (6392)	n/a	n/a

ATB, Antibiotics; POD, Postoperative day; TMP, Trimethoprim; TMP-SMX, Trimethoprim/sulfamethoxazole.

**Table 2:** Evaluation of study quality according to the Newcastle–Ottawa Scale.

Author/Year	Study Design	Selection (0–4)	Comparability (0–2)	Outcome (0–3)	Total NOS Score (0–9)
Meir 2004 [15]	Prospective cohort	3	1	2	6
Kanaroglou 2013 [16]	Prospective cohort	4	1	2	7
Zeiai 2016 [17]	Retrospective cohort	3	1	2	6
Roth 2018 [18]	Prospective RCT	4	2	3	9
Canon 2018 [19]	Retrospective comparative	3	1	2	6
Canon 2021 [20]	Retrospective comparative	3	1	2	6
Manchanda 2023 [21]	Prospective RCT	4	2	3	9
Doersch 2022 [22]	Retrospective comparative	3	1	2	6
Faasse 2022 [23]	Randomized double-blind trial	4	2	3	9
Basin 2025 [24]	Retrospective cross-sectional	3	1	2	6

NOS, Newcastle–Ottawa Scale; RCT, randomized controlled trial.

**Table 3:** Surgical outcomes between perioperative antibiotics vs. no antibiotics.

PERIOPERATIVE ANTIBIOTICS									
Author/Year	Series *n*=	Pre-op ATB (*n*= and Type)	Postop ATB (*n*= and Type)	Postop ATB Duration, Days (Range)	Infectious Complications, *n*= (%)	UCF *n*= (%)	Wound Dehiscence *n*= (%)	Meatal/Urethral Stenosis *n*= (%)	Other Complications *n*= (%)
Meir 2004 [15]	52	Cefonicid (52)	Cephalexin (52)	1 POD up to 2 days after stent removal	Bacteriuria: 11 (21.1) UTI: 3 (5.8)	3 (5.8)	0	0	Meatal regression: 1 (1.9)
	49	Cefonicid single dose (49)	n/a	n/a	Bacteriuria: 25 (51) UTI: 12 (24.5)	9 (18.4)	0	4 (8.2)	0
Kanaroglu 2013 [16]	78	Cefazolin single dose (78)	TMP (78)	1 POD for stenting duration	0	8/77 (10.4)	2/77 (2.6)	4/77 (5.2)	0
	71	Cefazolin single dose (71)	n/a	n/a	0	4/59 (6.8)	4/59 (6.8)	1/59 (1.7)	0
Zeiai 2016 [17]	58	TMP-SMX single-dose (58)	TMP/SMX (58)	1 POD up to 3–7 days after stent removal	3 (5.2)	5 (8.6)	6 (10.4)	1 (1.7)	3 (5.2)
	55	TMP-SMX single-dose (55)	n/a	n/a	WI: 1 (1.8) UTI: 1 (1.8)	2 (3.6)	5 (9.1)	0	1 (1.8)
Roth 2018 [18]	35	n/a	TMP-SMX (35)	6–10	WI: 2 (5.7) Bacteriuria: 2/28 (7.1)	1 (2.8)	1 (2.8)	1 (2.8)	0
Canon 2018 [19]	24	n/a	TMP-SMX (22) Nitrofurantoin (1) Cephalexin (1)	7.1	WI: 1 (4.2) UTI: 1 (4.2)	1 (4.2)	1 (4.2)	0	0
Canon 2021 [20]	159	Cefazolin or Clindamycin if penicillin allergy (159)	TMP-SMX or Nitrofurantoin/Cefalexin (159)	6.5 (5–8)	0	6 (3.8)	1 (0.6)	1 (0.6)	0
	64	Cefazolin or Clindamycin if penicillin allergy (64)	n/a	n/a	UTI: 1 (1.6)	2 (3.1)	0	0	0
	122	n/a	TMP-SMX or Nitrofurantoin/Cefalexin (122)	6.5 (5–8)	SSI: 1 (0.8)	9 (7.4)	0	3 (2.4)	Diverticulum: 1 (0.8)
Manchanda 2023 [21]	16	Ceftriaxone (16)	Ceftriaxone iv for 48 h, followed by oral Amoxiclav (16)	Stenting duration 7.5 (5–10)	Bacteriuria: 2 (12.5)	6 (37.5)	0	3 (18.7)	0
	24	Ceftriaxone (24)	n/a	n/a	Bacteriuria: 6 (25) SSI: 3 (12.5)	10 (41.7)	0	5 (20.8)	0
Doersch 2022 [22]	647	Cefazolin (634) Ampicillin (3) Cefoxitin (1) Ceftriaxone (1) Clindamycin (11) Gentamycin (3) Vancomycin (1)	Amoxicillin (12) Amoxi-clav (2) Cephalexin (276) Ciprofloxacin (1) Clindamycin (1) Nitrofurantoin (2) TMP (3) TMP-SMX (350)	Stenting duration 5 (3–7)	0	n/a	n/a	n/a	n/a
	80	Cefazolin (76) Clindamycin (4)	n/a	n/a	UTI: 6 (7.5) Cellulitis: 3 (3.7) Infected cyst: 1(1.2)	n/a	1 (1.2)	n/a	n/a
	34	n/a	Amoxicillin (3) Amoxi-clav (1) Clindamycin (4) TMP-SMX (25)	Stenting duration 5 (3–7)	0	n/a	n/a	n/a	n/a
Faasse 2022 [23]	45	n/a	TMP-SMX (45)	10	UTI: 2 (4.4) SSI: 1 (2.2)	5 (11.1)	1 (2.2)	1 (2.2)	Mild ADR: 3 (6.6)
Basin 2025 [24]	6392	Single dose (6392)	n/a	n/a	UTI: 50 (0.8)	n/a	164 (2.6)	n/a	n/a
**NO ANTIBIOTICS**									
Roth 2018 [18]	32	n/a	n/a	n/a	WI: 1 (3.1) Bacteriuria: 14/22 (63.6)	2 (6.2)	1 (3.1)	2 (6.2)	0
Canon 2018 [19]	24	n/a	n/a	n/a	0	1 (4.2)	1 (4.2)	1 (4.2)	0
Canon 2021 [20]	96	n/a	n/a	n/a	SSI: 1 (1.0)	3 (3.1)	2 (2.1)	2 (2.1)	0
Doersch 2022 [22]	67	n/a	n/a	n/a	UTI: 1 (1.5)	n/a	n/a	n/a	n/a
Faasse 2022 [23]	48	n/a	n/a	n/a	UTI: 3 (6.2) SSI: 1 (2.1)	1 (2.1)	0	0	Mild ADR: 5 (10.4)
Basin 2025 [24]	1221	n/a	n/a	n/a	UTI: 6 (0.5)	n/a	19 (1.5)	n/a	n/a

ATB, Antibiotics; UTI, Urinary tract infection; ADR, Adverse drug reaction; SSI, Surgical site infection; WI, Wound infection; UCF, Urethrocutaneous fistula; TMP, Trimethoprim; TMP-SMX, Trimethoprim/sulfamethoxazole; n/a, not/applicable.

## Data Availability

The data extracted from the included studies and used for all analyses in this systematic review are available from the corresponding author upon reasonable request. No analytic code was generated, as all analyses were performed using standard meta-analytic procedures implemented in Review Manager (RevMan) software (Version 5.4).

## Supplementary Materials

The following supporting information can be downloaded at: https://www.mdpi.com/article/10.3390/children13020194/s1, Figure S1: Funnel plots assessing publication bias for postoperative antibiotics alone versus no antibiotics, stratified by postoperative outcomes: (A) infectious complications, (B) urethrocutaneous fistula, (C) wound dehiscence, (D) meatal/urethral stenosis, and (E) other complications.; Figure S2: Funnel plots assessing publication bias for preoperative antibiotics alone versus no antibiotics, stratified by postoperative outcomes: (A) infectious complications and (B) wound dehiscence.; Figure S3: Funnel plot assessing publication bias for combined pre- and postoperative antibiotics versus no antibiotics, stratified by only (A) infectious complications.; Table S1: Full electronic search strategies.

## Abbreviations

The following abbreviations are used in this manuscript:

ADR	Adverse drug reaction
CI	Confidence interval
IRB	Institute Review Board
NOS	Newcastle-Ottawa Scale
OR	Odds ratio
PRISMA	Preferred Reporting Items for Systematic Reviews and Meta-analyses
RCT	Randomized controlled trial
SSI	Surgical site infection
TMP	Trimethoprim
TMP-SMX	Trimethoprim-sulfamethoxazole
UCF	Urethrocutaneous fistula
UTI	Urinary tract infection
WI	Wound infection
