# Supplementary material for: Do Perioperative Antibiotics Improve Outcomes After Hypospadias Repair? A Systematic Review and Meta-Analysis of Pediatric Literature
Source: Children (Basel). 2026 Jan 30;13(2):194. doi: 10.3390/children13020194 (PMC12939412; doi:10.3390/children13020194)
Supplement: Supplementary file 1 [file children-13-00194-s001.zip › children-4095801-supplementary - 副本/Table S1.docx]

**Supplementary Table S1. Full electronic search strategies.**

| **Database** | **Timeframe** | **Search strategy** |
| --- | --- | --- |
| PubMed / MEDLINE | 1 January 2000 – 31 March 2025 | (“Hypospadias”[MeSH] OR hypospadias[Title/Abstract]) AND (“Anti-Bacterial Agents”[MeSH] OR antibiotic*[Title/Abstract] OR antimicrobial*[Title/Abstract] OR prophylaxis[Title/Abstract]) AND (perioperative[Title/Abstract] OR preoperative[Title/Abstract] OR postoperative[Title/Abstract]) |
| Embase | 1 January 2000 – 31 March 2025 | ('hypospadias'/exp OR hypospadias:ti,ab) AND ('antibiotic agent'/exp OR antibiotic*:ti,ab OR antimicrobial*:ti,ab OR prophylaxis:ti,ab) AND (perioperative:ti,ab OR preoperative:ti,ab OR postoperative:ti,ab) |
| Scopus | 1 January 2000 – 31 March 2025 | TITLE-ABS-KEY (hypospadias) AND TITLE-ABS-KEY (antibiotic* OR antimicrobial* OR prophylaxis) AND TITLE-ABS-KEY (perioperative OR preoperative OR postoperative) |
| Web of Science | 1 January 2000 – 31 March 2025 | TS=(hypospadias) AND TS=(antibiotic* OR antimicrobial* OR prophylaxis) AND TS=(perioperative OR preoperative OR postoperative) |
