# Supplementary figures and images for: Do Perioperative Antibiotics Improve Outcomes After Hypospadias Repair? A Systematic Review and Meta-Analysis of Pediatric Literature
Source: Children (Basel). 2026 Jan 30;13(2):194. doi: 10.3390/children13020194 (PMC12939412; doi:10.3390/children13020194)

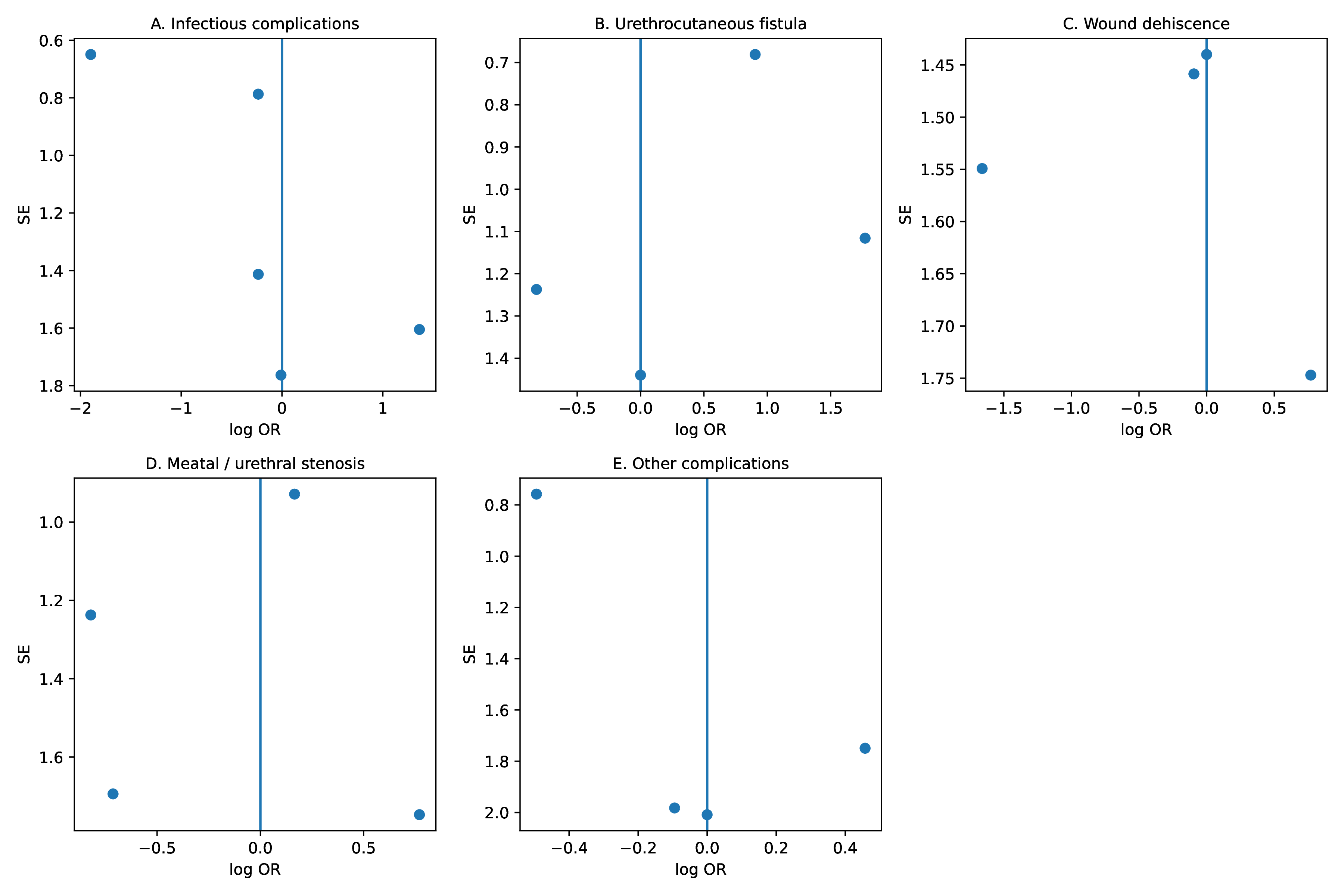

Supplement: Supplementary file 1 [file children-13-00194-s001.zip › children-4095801-supplementary - 副本/Supplementary Figure S1.tiff]

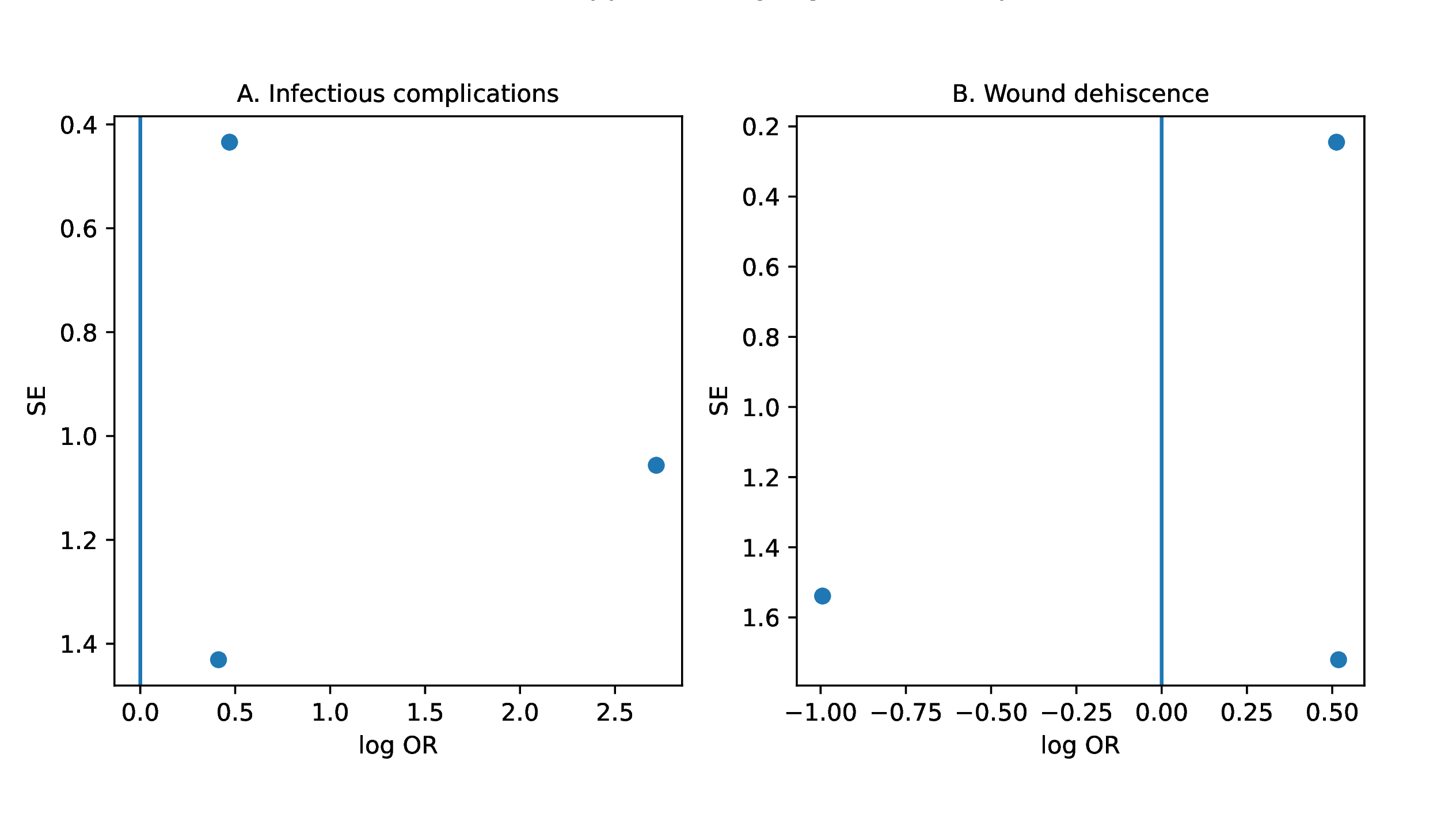

Supplement: Supplementary file 1 [file children-13-00194-s001.zip › children-4095801-supplementary - 副本/Supplementary Figure S2.tiff]

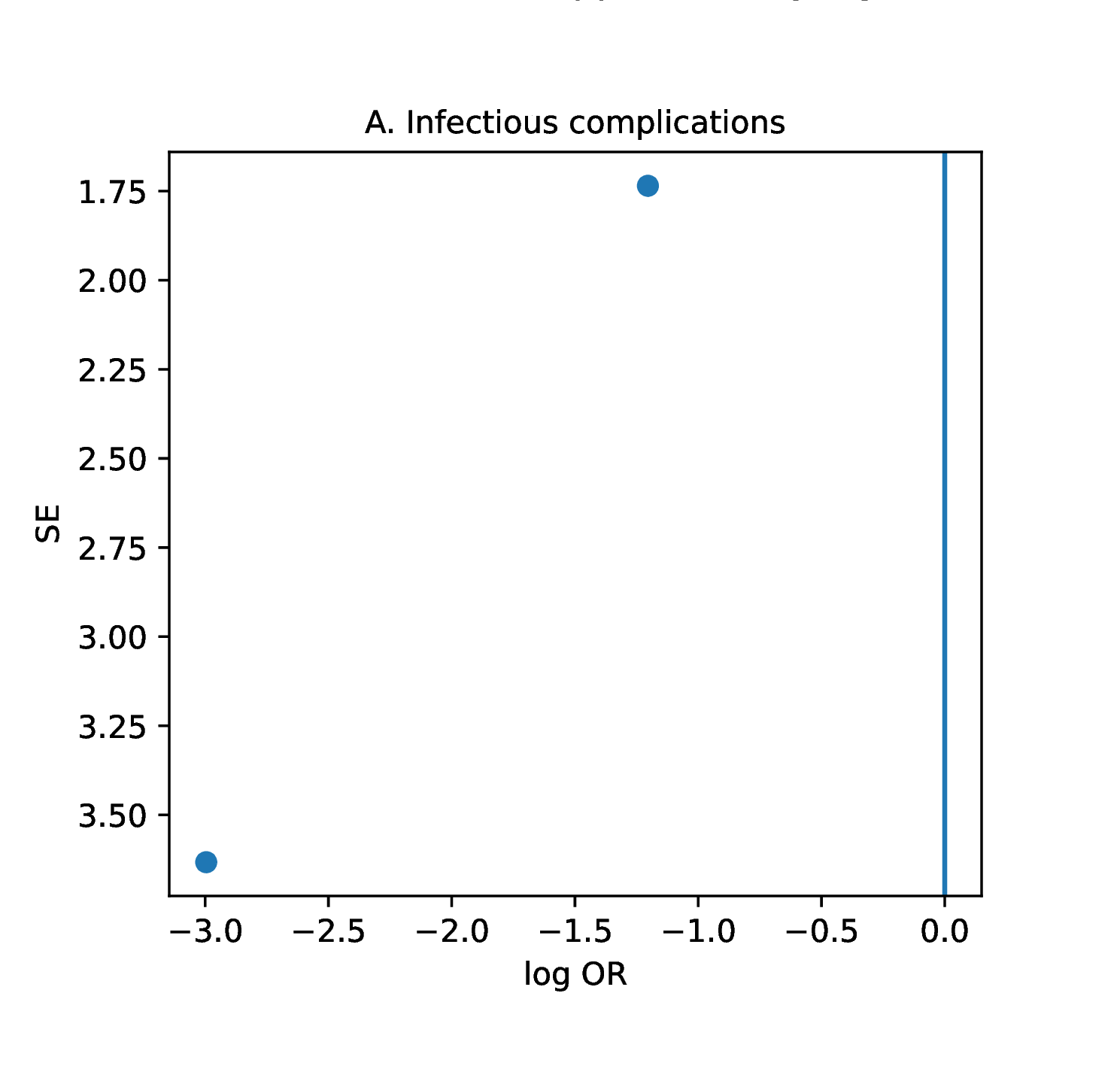

Supplement: Supplementary file 1 [file children-13-00194-s001.zip › children-4095801-supplementary - 副本/Supplementary Figure S3.tiff]
